# The Ever-Expanding Immunomodulatory Role of Calreticulin in Cancer Immunity

**DOI:** 10.3389/fonc.2015.00035

**Published:** 2015-02-20

**Authors:** Marco de Bruyn, Valerie R. Wiersma, Wijnand Helfrich, Paul Eggleton, Edwin Bremer

**Affiliations:** ^1^Department of Gynecologic Oncology, University Medical Center Groningen (UMCG), University of Groningen, Groningen, Netherlands; ^2^Department of Surgery, Translational Surgical Oncology, University Medical Center Groningen (UMCG), University of Groningen, Groningen, Netherlands; ^3^University of Exeter Medical School, Exeter, UK

**Keywords:** calreticulin, TNF, tumor necrosis factor related apoptosis inducing ligand, immunomodulation, immunogenic cell death, complex formation

## Abstract

Calreticulin is a pleiotropic molecule that normally resides in the lumen of the endoplasmic reticulum (ER). Here, it has various functions, ranging from regulation of calcium homeostasis to ensuring proper protein folding. More recently, calreticulin gained special interest for its extracellular functions, where it has direct immunomodulatory activity. In this respect, calreticulin activates dendritic cells and macrophages. In addition, certain anti-cancer therapies induce the translocation of calreticulin from the ER to the cell surface of dying cancer cells, where calreticulin dictates the immunogenicity of these cells. Interestingly, treatment with tumor necrosis factor (TNF)-related apoptosis inducing ligand (TRAIL) also induces membrane calreticulin exposure on cancer cells. As shown here, calreticulin directly interacts with TRAIL and its receptor-signaling complex, as well as with other TNF family members. Of note, TRAIL is a well known immunomodulatory molecule, and is expressed on the surface of natural killer T-cells. Therefore, calreticulin may have an as yet unrecognized wide(r) impact on immunity, with the TNF-ligand family modulating virtually all aspects of the immune response.

## Introduction

The endoplasmic reticulum (ER)-resident protein calreticulin is a pleiotropic molecule with many functions in the ER, ranging from protein folding, calcium homeostasis, and regulation of loading of antigenic peptides into major histocompatibility class I (MHCI) (1, 2). Thus, ER-resident calreticulin has an important impact on development and correct execution of immunity. However, in recent years, calreticulin has taken center stage not for its ER-related functions, but for its newly uncovered immunomodulatory effects in the extracellular space. It has become evident that ER chaperones such as calreticulin function as danger associated molecular pattern molecules (DAMPs) once outside the cell (3). For instance, when present on the cell surface of dendritic cells (DCs) calreticulin functions as a receptor for autocrine-produced complement factor 1q (C1q), which is upregulated during CD40L/CD40 signaling, a key T helper signal required for effective DC maturation (4). In addition, soluble calreticulin promotes differentiation of CD1dhi CD5^+^ B-cells into antibody-secreting cells (5). Further, soluble calreticulin induced TNF-α and IL-6 release from macrophages, which was regulated by scavenger receptor A (6).

A further prominent pro-immunogenic feature of extracellular calreticulin is its key role in the process of immunogenic cell death (ICD) (7). ICD is a type of apoptosis that is induced by certain chemotherapeutics or radiation, which promotes phagocytic uptake of apoptotic cells by professional antigen presenting cells, i.e., myeloid-derived DCs. Briefly, anthracycline or radiation therapy trigger the translocation of calreticulin to the pre-apoptotic tumor cell surface, which is dependent on the induction of an ER-stress response (8, 9). Cell surface exposed calreticulin then promotes clearance of the dying cancer cells by DCs, with subsequent release of ATP, HMGB1, and HSP70 from late apoptotic cells giving requisite cues for DC maturation and clonal T-cell expansion (10).

Immunogenic cell death has been described in a preclinical setting for an ever-expanding set of cytotoxic therapies, including many chemotherapeutics, radiation therapy, and photodynamic therapy. Interestingly, also treatment of cancer cells with a recombinant form of the tumor necrosis factor (TNF)-related apoptosis inducing ligand (TRAIL) was reported to induce calreticulin exposure and ICD (11). TRAIL is a member of the TNF superfamily that can bind to a set of five receptors; TRAILR1 (DR4), TRAILR2 (DR5), TRAIL-R3 (DcR1, TRID), TRAIL-R4 (DcR2, TRUNDD), and soluble osteoprotegerin. Binding of TRAIL to its agonistic receptors TRAILR1 and TRAILR2 induces assembly of the so-called death inducing signaling complex (DISC) to the intracellular death domain (DD). The DISC contains the adaptor molecule Fas-associated protein with death domain (FADD) and the pro-form of initiator caspase-8, which is auto-proteolytically processed in the DISC. Activation of caspase-8 triggers a proteolytic caspase cascade that ultimately leads to the execution of apoptosis. Of note, the pro-apoptotic signaling of the DISC can be inhibited by recruitment of additional regulators, such as cFLIP (12).

TNF-related apoptosis inducing ligand is an important immune effector molecule that is expressed on various types of immune cells including natural killer (NK) cells, T-cells, and natural killer T-cells (NKT cells) (13). Expression of TRAIL on T-cells is further increased during T-cell receptor (TCR) stimulation in the presence of interferon-γ (IFNγ) (14). Similarly, IFNγ-mediated activation of NK-cells, monocytes, and DCs enhances the expression of TRAIL on these cells (15, 16). TRAIL and its receptors (TRAILRs) play an important role in anti-tumor immunity, e.g., being involved in the immune surveillance for (metastatic) cancer cells by liver NK-cells (17). Thus, the induction of calreticulin exposure upon treatment with recombinant TRAIL may be related to a possible immunoregulatory effect of TRAIL in the context of its normal role on immune effector cells. Interestingly, calreticulin can also interact directly with TRAIL in a catching-type ELISA, as well as with the immunoregulatory TNF family members such as CD40 ligand (CD40L) and FasL (18). In contrast, calreticulin did not interact with TNF-α, adiponectin, or CD30L. The reported activation of ICD upon treatment with recombinant TRAIL suggests that calreticulin may regulate the pro-immunogenic effect of TRAIL during anti-tumor immunity.

## Calreticulin Directly Interacts with Pro-Apoptotic TNF-Ligands and Receptor Complexes

**Figure 1:**
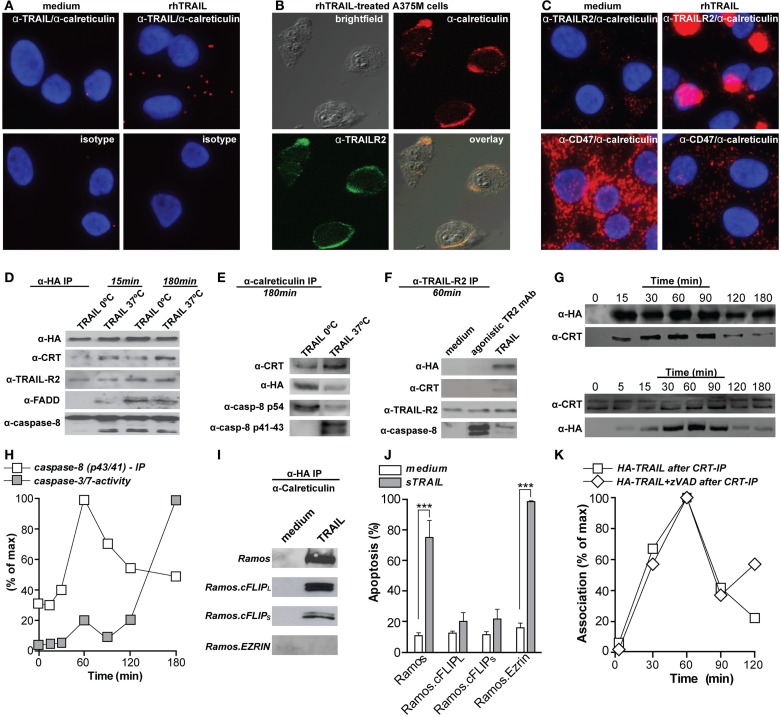
**Calreticulin binds to TRAIL and associates with the TRAILR2–DISC**. **(A)** A375M cells were treated with 100 ng/ml rhTRAIL for 1 h, incubated with mAbs directed against calreticulin (Ab2907, Abcam) and TRAIL (B-S23, Diaclone), or isotype controls, and subsequently analyzed by proximity ligation assay (Duolink II, Olink Biosciensce) (distance between both proteins; <40 nM or ~4–12 proteins). **(B)** A375M cells were treated with 100 ng/ml rhTRAIL for 1 h, double stained for calreticulin (red; Ab2907, Abcam) and TRAILR2 (green; HS-201, Alexis) and analyzed using confocal fluorescent microscopy. **(C)** As in **(A)**, but the proximity ligation assay were performed using mAbs directed against calreticulin (Ab2907, Abcam), TRAILR2 (HS-201, Alexis), and CD47 (ab3283, Abcam). **(D)** A375M cells were treated with HA-TRAIL [constructed by cloning the cDNA of HA-tagged TRAIL in frame into previously described vector pEE14 (23), yielding pEE14-HA-TRAIL] for 15 or 180 min at the indicated temperatures. Cells were subsequently lysed and HA-TRAIL was precipitated via affinity tag purification (HA-sepharose beads; 3F10, Roche). Precipitates were subsequently probed for the presence of HA (3F10, Roche), calreticulin (Ab39818, Abcam), TRAILR2 (HS-201, Alexis), FADD (272S, cell signaling), and caspase-8 (18C8, cell signaling). **(E)** As in **(D)**, but precipitated using anti-calreticulin. Precipitates were subsequently probed for the presence of calreticulin, HA, and caspase-8. **(F)** A375M melanoma cells were treated with HA-TRAIL or agonistic TRAILR2 mAb for 60 min at 37°C. Cells were subsequently lysed and TRAILR2 was precipitated. The precipitates were probed for the presence of HA, calreticulin, TRAILR2, and caspase-8. **(G)** A375M cells were treated with HA-TRAIL at 37°C for the indicated time-points. Cells were subsequently lysed and HA-TRAIL (upper panel) or calreticulin (lower panel) were precipitated. Precipitates were subsequently probed for the presence of calreticulin or HA-TRAIL, respectively. **(H)** A375M cells were treated with HA-TRAIL at 37°C for the indicated time-points. 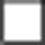 Quantification of caspase-8 (p42/41) association with TRAIL as determined by immunoprecipitation 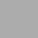 caspase-3/-7 activity as determined by flow cytometry (CellEvent caspase-3/-7 probe, Invitrogen). **(I)** Ramos, Ramos.cFLIP_L_, Ramos.cFLIP_S_, and Ramos.Ezrin cells were treated with HA-TRAIL for 1 h at 37°C. Cells were subsequently lysed and HA-TRAIL was precipitated via affinity tag purification. Precipitates were subsequently probed for the presence of calreticulin. **(J)** Ramos, Ramos.cFLIP_L_, Ramos.cFLIP_S_, and Ramos.Ezrin cells were treated with rhTRAIL for 16 h and apoptosis was assessed (DioC6 staining, flow cytometry using BD Accuri Flow cytometer). ***p < 0.001. **(K)** A375M cells were treated with HA-TRAIL in the presence or absence of 20 μM zVAD-fmk (CalBiochem). Subsequently, calreticulin was precipitated and the precipitates were stained for HA-TRAIL. Association of calreticulin and TRAIL is depicted as percentage of maximum association.

Calreticulin was recently reported to bind to TNF family member FasL in the synovial fluid of rheumatoid arthritis (RA) patients. This FasL/calreticulin interaction inhibited FasL-induced apoptosis of Jurkat T-cells (19). Since patients with RA have elevated levels of calreticulin in their serum, this might inhibit apoptosis of inflammatory T-cells in this particular disease. Our own studies confirm a similar direct interaction of calreticulin with TRAIL, as evidenced by, e.g., fluorescent microscopy for calreticulin and TRAIL on the cell surface of TRAIL-treated A375M melanoma cells and not in control cells (Figure 1A). This is in line with the reported translocation of calreticulin to the cell surface of apoptotic HeLa cells by recombinant TRAIL (11). Interestingly, calreticulin also strongly co-localized with TRAILR2 on TRAIL-treated A375M cells (Figures 1B,C), with calreticulin, TRAIL, and TRAILR2 all being in one complex co-precipitated upon anti-HA-TRAIL immunoprecipitation experiments (Figure 1D). Similarly, calreticulin was immunoprecipitated in the FasL/Fas DISC (data not shown). The association of calreticulin with TRAILR2 occurs in a clear patched pattern on the cell surface (Figures 1B,C), a pattern corresponding to that of the patched foci to which calreticulin redistributed on the cell membrane of apoptotic neutrophils (20), on mitoxantrone treated CT26 colon cancer cells (21), and hypericin-treated bladder cancer cells (22).

**Figure 2:**
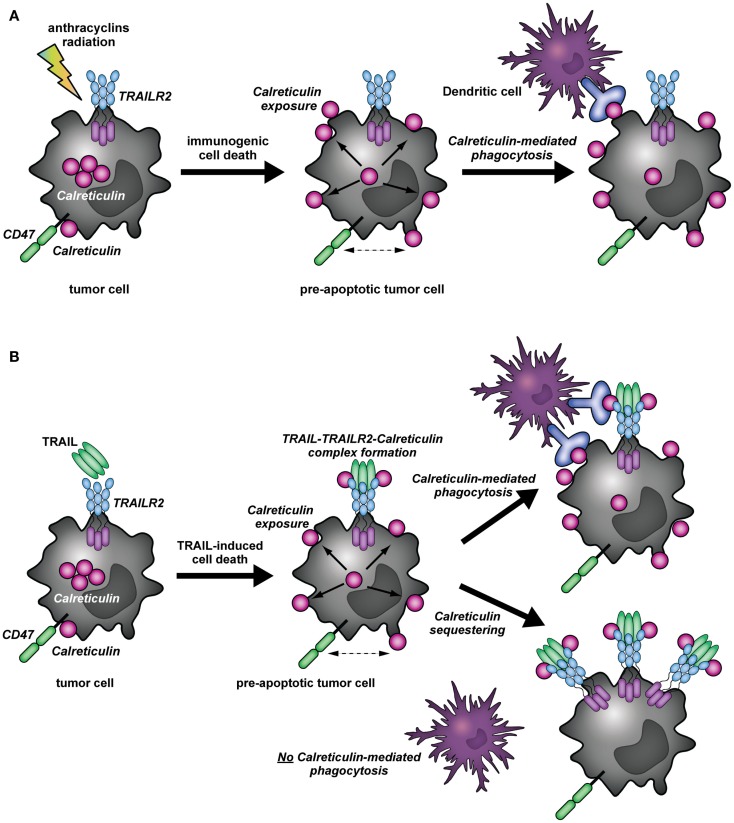
**Immunomodulatory role of calreticulin in cancer immunity (A)**. In (tumor) cells, calreticulin predominantly resides in the ER. Upon induction of immunogenic cell death (ICD) by, for instance, anthracycline or radiation therapy, calreticulin translocates to the surface of pre-apoptotic cells. In addition, surface exposed calreticulin (eat me signal) dissociates from CD47 (don’t eat me signal), whereupon the apoptotic cancer cells can be recognized and taken up by DCs. **(B)** The binding of TRAIL to its receptor (TRAILR2) results in the translocation of calreticulin to the cell surface of cancer cells, whereby a complex is formed between TRAIL, TRAILR2–DISC, and calreticulin. Simultaneously, calreticulin dissociates from CD47. The formation of the TRAIL/TRAILR2/Calreticulin-complex may have different outcomes: (1) As described for the concept of ICD, cell surface exposure of calreticulin and dissociation from CD47 induced by TRAIL treatment may facilitate phagocytic uptake by DCs. (2) the binding of calreticulin to TRAIL and TRAILR2 may impair phagocytic clearance by DCs, as calreticulin may be segregated away from the membrane microdomains in which it can partake in the phagocytic uptake by DCs.

The recruitment of calreticulin in distinct patches on the cell membrane has been shown to facilitate engulfment of apoptotic cells by phagocytes induced by UV radiation or anthracycline therapy (7, 20) (Figure 2A). These calreticulin-rich patches also contained high levels of phosphatidylserine (PS), an apoptotic marker that provides strong “eat me” signals for phagocytes (20). Correspondingly, calreticulin binds in a Ca^2+^-dependent manner directly to the polar head of PS with high affinity (*K*_D_ = 1.5 × 10^−5^ M) (24). This recruitment of calreticulin into lipid rafts was dependent on ERp57 (21, 25). Of note, on the surface of viable cells calreticulin is associated with CD47, a prominent so-called “don’t eat me” signal that blocks phagocytosis by binding to the inhibitory receptor SIRPα on phagocytes (26). During apoptosis, calreticulin dissociates from CD47 and associates with PS; a shift that promotes phagocytosis (20). Of note, the balance between pro-phagocytic calreticulin and anti-phagocytic CD47 has also been described in multiple human cancer cells, with cancer cells often being characterized by CD47 overexpression to elude phagocytic removal (26, 27). Similarly, our experiments indicate that calreticulin dissociates from CD47 upon TRAIL treatment of A375M cells (Figure 1C). Together with literature, this dissociation suggests that the interaction of calreticulin with TRAIL and TRAILR2 may facilitate phagocytic clearance (Figure 2B).

## Calreticulin is Recruited to Active Signaling Complexes of the TNFR Superfamily

In an earlier study on TNFR-signaling, surface expressed calreticulin was reported to be recruited to TNF receptor 1 (TNFR1) and its adaptor TNFR-associated death domain (TRADD) upon treatment with bacterial peptidoglycan *N*-acetylmuramyl-l-alanyl-d-isoglutamine (l,d-MDP) (28). This interaction was independent of TNF and triggered apoptosis in RK_13_ kidney cells that could be blocked by anti-calreticulin antibodies. Similarly, calreticulin associated with TRAIL, TRAILR2, as well as adaptor protein FADD and pro-caspase-8 in A375M melanoma cells (Figures 1D,E). Of note, caspase-8 was predominantly present in its pro-form after 15 min of TRAIL treatment, but was in its processed p41/43 form after 180 min. Further, caspase-8 processing occurred mainly at 37°C, although treatment on ice also induced DISC formation (Figures 1D,E). The interaction of calreticulin with TRAIL was necessary for its association with TRAILR2 as calreticulin was not co-immunoprecipitated with TRAILR2 upon treatment with agonistic TRAILR2 antibody, despite DISC formation (Figure 1F). Thus, physical interaction of TRAIL with calreticulin mediates its recruitment to the TRAILR2 DISC.

## Calreticulin Association with the TRAILR2–DISC Depends on Raft Reorganization but is Independent of Apoptotic Signaling

During ICD with mitoxantrone, the maximum level of surface exposed calreticulin was observed within 15 min, and remained at stable levels for at least 24 h (7). In line with this, surface calreticulin became visible after 30 min on neutrophils and continued to increase as neutrophils became more apoptotic (20). In contrast, induction of ICD upon treatment of Hela cells with TRAIL only induced detectable levels of calreticulin on the surface after 24 h of incubation (11). On A375M cells, calreticulin translocated to and associated with calreticulin with TRAIL already after 15 min, with maximal association after 1 h (Figure 1G), which corresponded to the maximum level of processed caspase-8 (p43/41) (Figure 1H). Further, the maximum association of calreticulin with TRAIL and the DISC occurs before the execution of apoptosis, as determined by activation of effector caspases-3 and -7 (Figure 1H). This dynamic corresponds to the pre-apoptotic exposure of calreticulin during ICD (7). Interestingly, induction of apoptosis was required to induce cell surface calreticulin exposure upon treatment with mitoxantrone, with depletion of caspase-8 completely abolishing the translocation of calreticulin (11). In contrast, inhibition of caspase-8 did not inhibit calreticulin exposure upon photodynamic therapy with hypericin (9). In line with the latter report, calreticulin was still co-precipitated with TRAIL in Ramos B-cells that are resistant to TRAIL-mediated apoptosis by overexpression of the anti-apoptotic DISC component cFLIP (Ramos.cFLIP_L_ and Ramos.cFLIP_s_) (Figures 1I,J). Correspondingly, co-treatment of A375M cells with TRAIL and pan-caspase-inhibitor zVAD-fmk did not abrogate the association of calreticulin with TRAIL (Figure 1K).

During ICD, the actin cytoskeleton was required for translocation of calreticulin to the pre-apoptotic cell surface (11). Similarly, calreticulin–TRAIL association was dependent on cytoskeletal rearrangements and was abolished in Ramos cell overexpressing the double negative Ezrin mutant (DN-Ezrin), which is incapable of interacting with actin (Figure 1I). Thus, calreticulin association with the DISC is independent of apoptosis, but dependent on cytoskeletal reorganization.

## Conclusion/Future Directions

It is clear from literature that treatment of cancer cells with TRAIL can induce calreticulin relocalization to the cell surface. Further, as evidenced by the data in the current manuscript, surface calreticulin is recruited to the TRAIL/TRAILR/DISC complex and dissociates from CD47. Together, these observations suggest that calreticulin could promote phagocytosis of apoptotic cells upon TRAIL-mediated killing (Figure 2B). On the other hand, its association with the TRAILR2–DISC might render calreticulin unable to effectuate pro-immunogenic removal of target cells by DCs. In this respect, it is tempting to speculate that by binding to the TRAILR2–DISC, calreticulin is segregated away from the membrane microdomains in which it can partake in the phagocytic uptake of target cells by DCs or other phagocytes (Figure 2B). These findings would be consistent with the non-immunogenic clearance of cells undergoing TRAIL-mediated programed cell death (29). To gain insight in the possible immunomodulatory role of calreticulin during TRAIL-induced apoptosis, it will be imperative to characterize the particular complex or membrane microdomain in which calreticulin resides in the context of ICD. Comparison of calreticulin localization upon treatment with typical immunogenic inducers, and how this compares to its localization during TRAIL-mediated cell death may shed light on its particular role.

The inhibition of FasL-mediated T-cell death by calreticulin, the direct induction of apoptosis by l,d-MDP-bound calreticulin via TNFR1 and the here described association of calreticulin to TRAILR2 during TRAIL-mediated cell death suggest a much broader role for calreticulin in the antitumor immune response than as a mediator of ICD. By directly modulating apoptosis and associating with key apoptotic players, calreticulin could function as a bridge between innate and adaptive immunity. In this paradigm, induction of programed cell death by immune effector molecules, such as TRAIL and FasL, induces a rapid pre-apoptotic translocation of calreticulin to the cell surface. On the cell surface, calreticulin can then either modulate induction of cell death as described for FasL (19) or translocate to specific cell surface microdomains (with or without receptors of the TNF superfamily), thereby modulating phagocytosis and pro-immunogenic DC-mediated removal. In its soluble state, secretion of calreticulin in response to apoptotic stimuli can directly affect the immediate early innate immune response by modulating cytokine secretion of macrophages. Interestingly, agonistic TRAIL-receptors have been reported as negative regulators of innate immune responses (30). An intriguing possibility currently being investigated in our laboratory is that sequestration of calreticulin by TRAIL-receptors could contribute to limiting activation of innate immunity.

Calreticulin exposure can further trigger activation of the adaptive immune response by promoting immunogenic DC-uptake of apoptotic cells during ICD. In this respect, it is also of interest that of all the TNF ligands it was found to interact with in a catching-type ELISA, calreticulin most strongly associated with CD40L. Thus, recruitment of calreticulin to CD40L/CD40 signaling complexes may impact DC activity. Whether calreticulin directly associates with TNF family members beyond CD40, FasL, and TRAIL has not been identified, to date. However, the reported interaction of calreticulin with several TNF-ligand family members points to the possibility that calreticulin may have an as yet unrecognized wide(r) impact on immunity, with the TNF ligand family modulating virtually all aspects of the immune response.

## Conflict of Interest Statement

The authors declare that the research was conducted in the absence of any commercial or financial relationships that could be construed as a potential conflict of interest.
